# Identification of a Novel Prognostic Signature for Gastric Cancer Based on Multiple Level Integration and Global Network Optimization

**DOI:** 10.3389/fcell.2021.631534

**Published:** 2021-04-12

**Authors:** Lin Cui, Ping Wang, Dandan Ning, Jing Shao, Guiyuan Tan, Dajian Li, Xiaoling Zhong, Wanqi Mi, Chunlong Zhang, Shizhu Jin

**Affiliations:** ^1^Department of Gastroenterology and Hepatology, The Second Affiliated Hospital, Harbin Medical University, Harbin, China; ^2^Department of Interventional Radiology, The Third Affiliated Hospital, Harbin Medical University, Harbin, China; ^3^College of Bioinformatics Science and Technology, Harbin Medical University, Harbin, China; ^4^Department of Gastroenterology and Hepatology, The First Hospital Of Harbin, Harbin, China

**Keywords:** gastric cancer, integrated analysis, competing endogenous RNAs, random walk algorithm, prognostic signature

## Abstract

Gastric Cancer (GC) is a common cancer worldwide with a high morbidity and mortality rate in Asia. Many prognostic signatures from genes and non-coding RNA (ncRNA) levels have been identified by high-throughput expression profiling for GC. To date, there have been no reports on integrated optimization analysis based on the GC global lncRNA-miRNA-mRNA network and the prognostic mechanism has not been studied. In the present work, a Gastric Cancer specific lncRNA-miRNA-mRNA regulatory network (GCsLMM) was constructed based on the ceRNA hypothesis by combining miRNA-target interactions and data on the expression of GC. To mine for novel prognostic signatures associated with GC, we performed topological analysis, a random walk with restart algorithm, in the GCsLMM from three levels, miRNA-, mRNA-, and lncRNA-levels. We further obtained candidate prognostic signatures by calculating the integrated score and analyzed the robustness of these signatures by combination strategy. The biological roles of key candidate signatures were also explored. Finally, we targeted the PHF10 gene and analyzed the expression patterns of PHF10 in independent datasets. The findings of this study will improve our understanding of the competing endogenous RNA (ceRNA) regulatory mechanisms and further facilitate the discovery of novel prognostic biomarkers for GC clinical guidelines.

## Introduction

Gastric Cancer (GC) is the second leading cause of cancer death globally according to the latest WHO statistics in 2018 (Bray et al., 2018). Early GC can be removed by Endoscopic Mucosal Resection or Endoscopic Submucosal Dissection, and the long-term prognosis is good at present (Ko et al., 2016). However, GC is usually diagnosed at an advanced stage when it has spread to other parts of the body, the 5-year survival rate for patients with GC remains low (Gong et al., 2019). Therefore, how to reduce this clinical threat to human survival and identify prognostic signatures are of great importance for the treatment of GC patients.

There is growing evidence that non-coding RNAs (ncRNAs), which make up the majority of human RNAs, play key roles in regulating gene expression, though they are not translated into proteins (Kaikkonen et al., 2011; Zhang et al., 2019). MicroRNA (miRNA) is one type of ncRNA that contains approximately 22 nucleotides. The expression profiling of miRNA has attracted extensive attention because of its important role in the proliferation, differentiation, apoptosis, and other biological processes of cancer cells (Ling et al., 2013). Some miRNAs may serve as an indicator of poor survival for cancer patients (Lan et al., 2015). Long non-coding RNAs (lncRNAs) are defined as ncRNAs over 200 nucleotides in length (Ransohoff et al., 2018). More evidence has revealed that lncRNAs can regulate the expression of protein-coding genes at the epigenetic, transcriptional, and posttranscriptional levels, with prototypes including scaffolds, signals, guides, and decoys (Chew et al., 2018; Li et al., 2019; Liu et al., 2019). Dysregulation of lncRNA expression has been documented in a variety of diseases, especially in cancers (Prensner and Chinnaiyan, 2011).

Up to now, the prognostic signatures of multiple genes and ncRNAs have been identified for GC by using high-throughput expression profiling. CCNB1, PLK1, and PTTG1 have been identified as new prognostic markers and targets in the GC treatment (Weichert et al., 2006; Dibb et al., 2015; Wang et al., 2015; Xu et al., 2016). Through bioinformatics analysis and *in vitro* experiments, FKBP10 was verified to be a novel biomarker of prognosis and lymph node metastasis (LNM) in GC (Liang et al., 2019). In terms of ncRNAs, a previous study found that a 4-miRNA (miR-128, miR-27b, miR-214, and miR-100) signature predicted the occurrence of LNM after endoscopic submucosal dissection, suggesting that patients with higher scores tended to have higher LNM than patients with lower scores (Liu et al., 2017). A signature comprised of three miRNAs in serum can predict the clinical outcome for advanced GC. As a supplement to the TNM staging system, postoperative risk stratification can be improved (Chen et al., 2020). A lncRNA-based signature was identified from Gene Expression Ominus (GEO) datasets to contribute to the prognosis and predictive personalization of GC and served as potential GC biomarkers (Zhu et al., 2016).

The competing endogenous RNA (ceRNA) activity is widely recognized as an important regulatory mechanism for functional lncRNAs. In the ceRNA hypothesis, lncRNAs could communicate with mRNAs *via* serving as miRNAs sponges to reduce the miRNA-targeting inhibition on mRNAs (Ala, 2020). Interestingly, more studies have shown that this regulatory map exists in GC. For instance, lncRNA CCDC144NL-AS1 sponges miR-143-3p and acts as a ceRNA to regulate MAP3K7 in GC (Fan et al., 2020). In addition, LncRNA LINC00689 up-regulates ADAM9 through sponging miR-526b-3p, promoting GC progression. Moreover, lncRNA LINC00689 promotes the progression of GC by upregulation ADAM9 through sponging miR-526b-3p (Fan et al., 2020). MiRNAs influence biological processes by negatively regulating mRNA expression levels, thereby mediating the pathway activities. High-throughput assay of ncRNA or mRNA expression allows for the simultaneous measurement of a large number of ncRNAs or mRNAs, which is an important resource in tumor biology. High-throughput expression datasets and the detailed clinical information of corresponding patients can be obtained from some databases, such as The Cancer Genome Atlas (TCGA) (Fang and Fullwood, 2016).

In the present work, a Gastric Cancer specific lncRNA-miRNA-mRNA regulatory network (GCsLMM) was constructed based on the ceRNA hypothesis, by combining miRNA-target interactions and expression data sets of GC. To mine the novel prognostic signatures related to GC, we performed a random walk with a restart algorithm based on the GCsLMM from three levels (miRNA, lncRNA, and mRNA). Based on the optimization results, we further analyzed the lncRNA-miRNA-mRNA combination signatures and confirmed the prognostic performance of the top combinations. Finally, we focused on a novel gene, PHF10. The expression pattern of PHF10 was explored and validated in online websites and independent datasets. In conclusion, this study will improve our understanding of the complex regulatory mechanisms in GC prognosis, and further facilitate the discovery of novel prognostic biomarkers for patient treatment.

## Materials and Methods

### Expression Data Sources

The training datasets, including the expression datasets and the patients’ clinical information, were downloaded from the TCGA database^1^. The mRNA/lncRNA expression was generated by HTseq-FPKM and miRNA expression was generated by miRNA-seq-BCGSC-miRNA Profiling analysis. The expression values (FPKM) of RNA from each sample were obtained, and the average expression values were used as the final values for the repeated samples. In addition, samples with survival time less than 30 days were excluded because these patients may have died of other causes. Finally, the datasets used in this study included the expression profiles of RNAs (including mRNAs, lncRNAs, and miRNAs), as well as patients’ clinical information.

To test the predictive power of the PHF10 signature, we obtained another GC mRNA expression dataset (access no: GSE38749, platform: GPL570) from the GEO database^2^. This dataset included 15 GC samples and corresponding clinical information was also obtained.

### Protein-Protein and miRNA-Target Interactions

The protein-protein interaction (PPI) network was obtained from a previous study (Cheng F. et al., 2019) that used 12 common databases, including BioGRID, DFCI_NET_2016, HI-II-network, HPRD, InnateDB, INstruct, IntAct, KinomeNetworkX, MINT, PhosphositePlus, PINA, and SignaLink2.0. To make further analysis more robust, the PPI interactions found in at least two databases were considered to be ultimate PPI networks, with a total of 12,512 protein nodes and 83,065 interactions.

Data of miRNA-mRNA interactions were downloaded from four of the most commonly used databases, including the miRTarBase, miR2 Diseases, miRecords, and TarBase. From these databases, we obtained 6,459 miRNA-mRNA interactions with 358 miRNAs and 3,452 target mRNAs from low-throughput trials. Data of miRNA-lncRNA interactions were downloaded from the TarBase database. Finally, we integrated PPI relation and miRNA-target (including miRNA-mRNA and miRNA-lncRNA) as candidate complex networks.

### Screening GC Specific Survival-Related RNA Relations

Based on the clinical information of the GC samples from TCGA, we first utilized the median survival time as a cutoff to define prognostic groups with good and poor outcomes. Within these two groups, we then calculated the Pearson correlation between all RNA relations (gene-gene, miRNA-mRNA, and miRNA-lncRNA) from the complex network mentioned above, respectively. If the RNA pairs displayed positive or negative correlations in the group with good prognosis, displayed the opposite directions (negative or positive) or no significant results in another group. Then, this kind of RNA pair was considered as GS survival-related relations. All these RNA pairs from the global network were further selected as GCsLMM.

### Network-Based Random Walk Algorithm

Based on the GCsLMM network, we further performed a global risk impact analysis to optimize prognostic signatures by using the random walk with restart algorithm (Kohler et al., 2008). We performed random walk analysis to optimize the corresponding prognostic signatures from miRNA-, mRNA-, and lncRNA-levels, respectively. Take the miRNA-level as an example, we first identified prognostic miRNAs using the univariate cox method. Then, we annotated prognostic miRNAs into the GCsLMM network and regarded these miRNA nodes as seeds.

The random walk algorithm was finally used to evaluate the global risk impact of prognostic miRNAs on all components within the network as follows:

$$P^{t+1}=\left(1-r\right)\,W\,P^{t}+r\,P^{0}$$

where *W* was the column-normalized adjacency matrix of the GCsLMM network, which consisted of 0 and 1.*P*^*t*^ was a vector, in which a node in the global network held the probability of finding itself in this process up to step *t*. The initial probability vector *P*^0^, was constructed in such a way, where equal probabilities were assigned to all seed miRNAs and the sum of their probabilities was equal to 1. Additionally, the restart of the walker at each step was the probability, *r*.*r* = 0.7). When the difference between *P*^*t*^ and *P*^*t* + 1^fell below 10^–6^, the probabilities reached a steady state. Finally, each component in the network was given a score according to the values in the steady-state probability vector, *P*^∞^from the miRNA-level random walk. A similar procedure was also performed at mRNA and lncRNA levels with prognostic mRNA and lncRNAs as seed nodes.

### Enrichment Analysis

Gene Ontology (GO) and KEGG enrichment analyses were performed using the R *clusterProfiler* package. In this study, we performed two kinds of enrichment analyses: (i) enrichment analysis for protective and risk mRNAs from univariate cox analysis using the overlapping method, and (ii) enrichment analysis for all the ranking mRNAs obtained from co-expression analysis with candidate top signatures (miR-22, PHF10, and LINC00592) using the Gene Set Enrichment Analysis (GSEA) method. In all enrichment analyses, the adjusted *P*-values < 0.05 were considered as significant results.

## Results

### The Workflow of This Study

**FIGURE 1:**
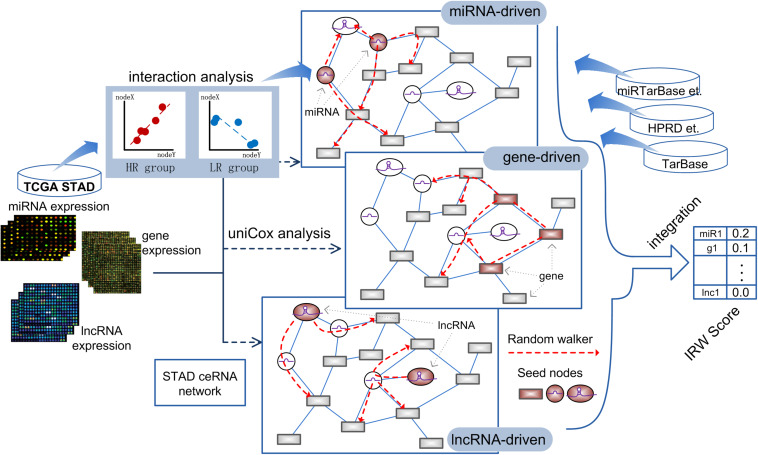
The workflow of calculating IRW score.

Based on the high-throughput expression datasets of GC from TCGA and miRNA-target interaction resources, we constructed a GC specific network named GCsLMM and calculated the integrated score (Integrated Random Walk, IRW) for each candidate signature in three steps. The detailed workflow was displayed in Figure 1.

Step 1, we respectively obtained the gene-gene, miRNA-mRNA, and miRNA-lncRNA interactions from public databases to form a global network (see section “Materials and Methods”). Furthermore, we screened the survival-related RNA regulations to construct a GC survival specific network, GCsLMM (see section “Materials and Methods”).

Step 2, based on the expression matrix and clinical information of GC, we respectively identified GC prognostic related mRNAs, miRNAs, and lncRNAs by using the univariate cox method.

Step 3, we used a network-based random walk algorithm to evaluate the prognostic effects from three levels (see section “Materials and Methods”). Finally, we calculated the IRW score, which was the mean score from three level random walk analyses for each molecule from GCsLMM. The molecules with higher IRW scores were regarded as candidate prognostic signatures.

### Calculating IRW Score for Screening Candidate Prognostic Signatures

**FIGURE 2:**
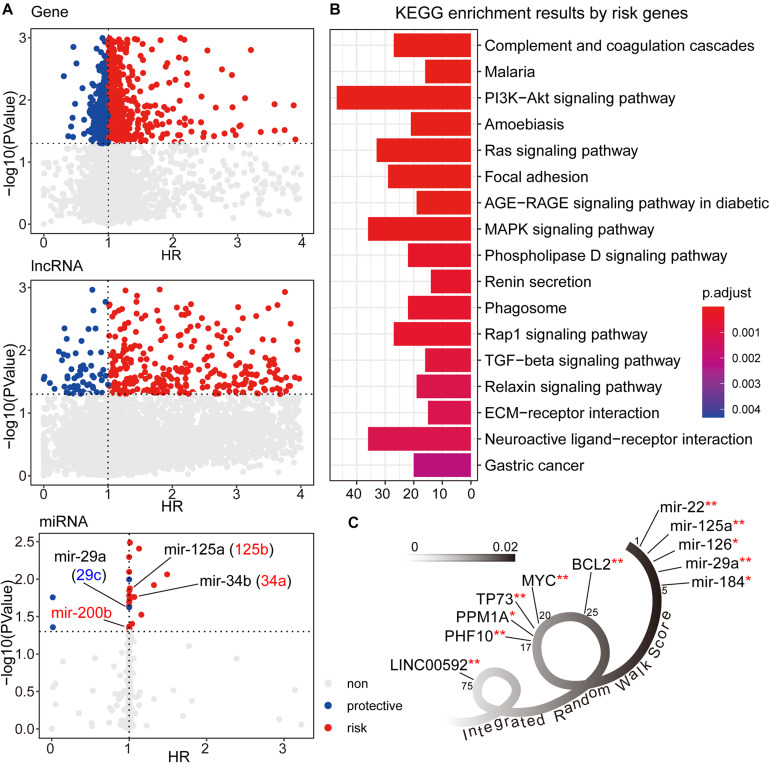
**(A)** The univariate cox results for prognostic genes, lncRNAs, and miRNAs. **(B)** The KEGG enrichment analysis results for risk genes from univariate cox results. The corresponding enrichment results for protective genes were none. **(C)** The candidate signature results with decreasing IRW score. **symbol indicated the GC related markers, and *symbol indicated tumor related markers.

We first used the univariate cox method to calculate the associations between RNA expression and GC patient prognosis with *P*-value < 0.05. Considering the HR values from cox results, we further defined the prognostic factors as protective factors with HR < 1 and risk factors with HR > 1 (see Figure 2A). In total, we identified 1,405 prognostic mRNAs (903 risk, 502 protective), 1,151 prognostic lncRNAs (1,090 risk, 61 protective), and 28 prognostic miRNAs (22 risk, six protective). To dissect the functional roles of the prognostic factors at mRNA level, we respectively performed the GO and KEGG enrichment analyses for protective mRNAs and risk mRNAs (see Figure 2B and Supplementary Figure 1). A non-significant result was obtained from protective mRNAs. For the risk genes, we identified many cancer related pathways, such as “PI3K-Akt signaling pathway,” “MAPK signaling pathway,” “TGF-beta signaling pathway,” and “ECM receptor interaction,” which were closely associated with the biological mechanism of GC. At the miRNA level, many of the prognostic miRNAs we identified performed important roles involved in GC, such as miR-29a, miR-125a, and miR-34b.

After three-level random walk analyses, we calculated the IRW score for each molecule within the GCsLMM. Most of the candidate signatures with a higher score displayed close associations with GC or other tumors (see Figure 2C). The signature with the highest IRW score was miR-22, and the up-regulation of miR-22-3p has been confirmed as related to GC cell proliferation inhibition and apoptosis process (Gan et al., 2019). For the gene results, the top five genes (ranked from 17-20 and 25) were also associated with tumor formation and prognosis, including TP73 (Zhang et al., 2018) and MYC (Calcagno et al., 2008). Other evidence has revealed that LINC00592 (top one lncRNA, ranked 75) was included in lncRNA biomarkers which were associated with disease free survival in the patients of GC (Cheng C. et al., 2019).

### The Survival Predictive Power of lncRNA-miRNA-mRNA Combinations

**FIGURE 3:**
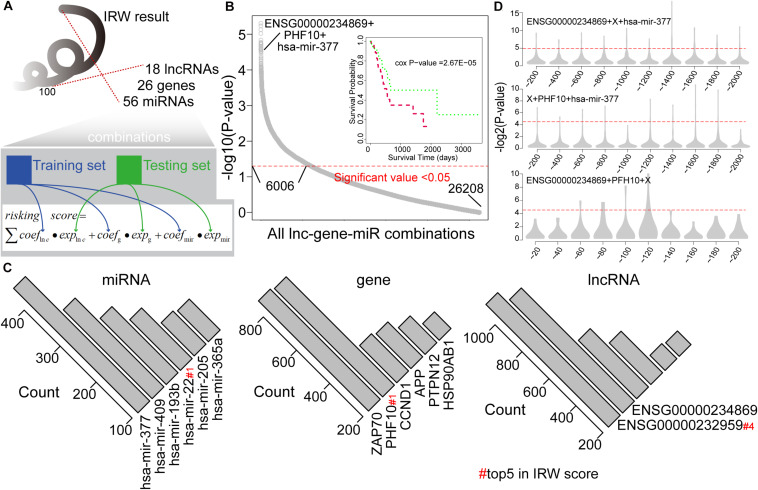
**(A)** The framework for constructing all lncRNA-gene-miRNA combinations and calculating the risk score based on each combination. **(B)** Distribution of prognostic related combinations within all combinations. A K-M plot, for example, ENSG00000234869 + PHF10 + hsa-mir-377. **(C)** The robust signatures for miRNA, gene, and lncRNA within all prognostic related combinations. **(D)** Random results when changing each molecule from the example combination in label **(B)** by molecules with low IRW scores.

We then explored the prognostic power of combinations of lncRNAs, miRNAs, and mRNAs with high IRW scores, and explored the robustness of these signatures. Based on the IRW score, we first constructed 26,208 combinations that contained one lncRNA, one gene, and one miRNA, using the top 100 molecules (18 lncRNAs, 26 genes, and 56 miRNAs). Then, we divided the total training expression matrix into two independent data sets, inner training set, and inner testing set. The inner training set was used to calculate the univariate coefficient for each component within the combination, and the inner testing set was used to calculate the prognostic performance (*p*-value) of the combination signature (see Figure 3A). As a result, a total of 6,006 combinations displayed significant results with *P*-value < 0.05 (see Figure 3B). Furthermore, we undertook a statistical analysis of how many times each molecule was included in the significant combinations. As shown in Figure 3C, miR-377 and miR-409 were the most robust prognostic miRNAs, and miR-22 with the highest IRW score also ranked fourth. At the gene level, ZAP70 and PHF10 were the most robust signatures. ENSG00000232959 and ENSG00000234869 were the most robust prognostic lncRNAs. From these robust prognostic signatures, the combination (ENSG00000234869-PHF10-miR-377) displayed significant predictive power with *P*-value = 2.67E-05.

To further test the performance of robust signatures, we took the ENSG00000234869-PHF10-miR-377 signature as an example and performed perturbation analysis as follows. For this combination signature, we respectively exchanged the lncRNA, miRNA, or gene molecule and kept the other two molecules unchanged. Corresponding molecules were exchanged as N molecules with the lowest IRW score (*N* = 100 for gene, *N* = 100 for lncRNA, and *N* = 20 for miRNA). Then, based on new combination signatures with random analysis, we developed a new score model for patient risk evaluation. Finally, the inner testing set was also used to calculate the significance of random combinations. As shown in Figure 3D, the random combinations displayed worse predictive performance than previous robust signatures, which confirmed that our signatures are more robust with a high IRW score than random results.

### The Functional and Network Analysis for miR-22, PHF10, and LINC00592

**FIGURE 4:**
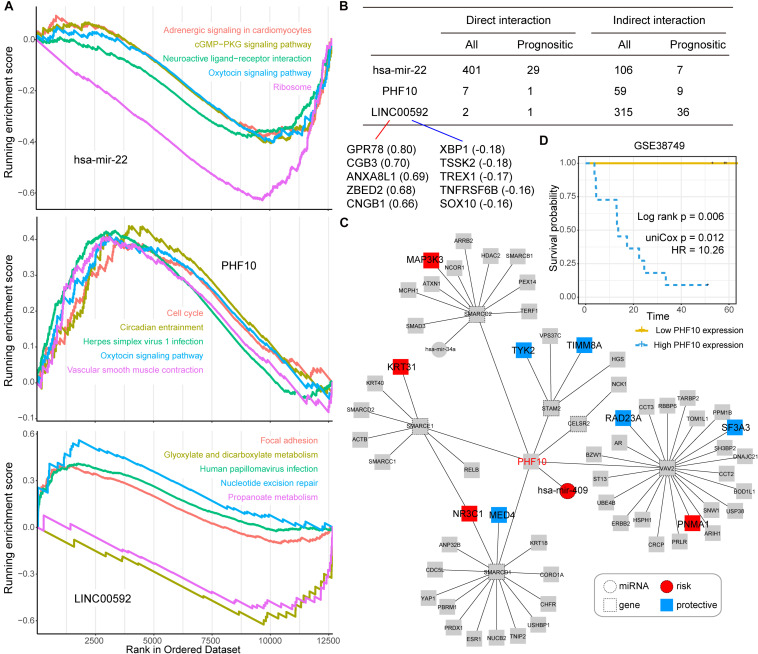
**(A)** The top five Gene Ontology (GO) terms for three candidate signatures, hsa-mir-22, PHF10, and LINC00592 using the GSEA method. **(B**) The prognostic statistic information of direct or indirect interaction of three candidate signatures, hsa-mir-22, PHF10, and LINC00592 within the global network. **(C)** The PHF10 core network. Red node indicates risk signatures, and blue node indicates protective signatures. **(D)** The K-M plot of PHF10 expression in GSE38749 and median expression level of PHF10 was the cut-off to define two groups, and the log-rank test was used to calculate the *P*-value.

Next, we took three candidate prognostic signatures (miR-22, PHF10, and LINC00592) with the highest IRW score from each level to explore their biological mechanisms. Based on the TCGA expression datasets, we calculated the expression correlations between each of the candidate signatures and total mRNAs. All mRNAs were ranked in descending order after correlation analysis and GSEA enrichment analysis was performed (see section “Materials and Methods”). As shown in Figure 4A, miR-22 was negatively enriched in the “cGMP-PKG signaling pathway,” “Oxytocin signaling pathway,” and “Ribosome.” The PHF10 was positively enriched in the “Cell cycle” and “Oxytocin signaling pathway.” The lncRNA LINC00592 was positively enriched in “Focal adhesion,” and negatively enriched in “Glyoxylate and dicarboxylate metabolism” functions.

Most of the direct neighbors of these three signatures were related to GC prognosis. As shown in Figure 4B, 29 of the miR-22’s 401 direct neighbors were prognostic related factors, and seven indirect neighbors which connected the miR-22 by another node were also prognostic related. For LINC00592, there were two direct neighbors, of which one was prognostic, and 36 of the 315 indirect neighbors were prognostic. Notably, miR-22 and LINC00592 were significant prognostic factors with univariate *P*-value = 0.0079 and 0.0002, however, PHF10 is not a significant prognosis factor. We therefore further displayed the interaction network of PHF10 (see Figure 4C). In this network, the miR-409 which has a regulatory role on PHF10 was a risk factor. Wei et al. (2010) found that PHF10 inhibited the expression of caspase-3 and damaged the programmed cell death pathway in human GC. By targeting PHF10 in GC, microRNA-409-3p was also verified to suppress cell proliferation and apoptosis (Li et al., 2012). Furthermore, the predictive power of PHF10 was tested in another independent data set, GSE38749. As shown in Figure 4D, patients with a higher expression of PHF10 exhibited a lower survival rate than patients with lower expression, showing its risk roles.

### The Expression Pattern of PHF10 in GC Formation and Clinical Events

**FIGURE 5:**
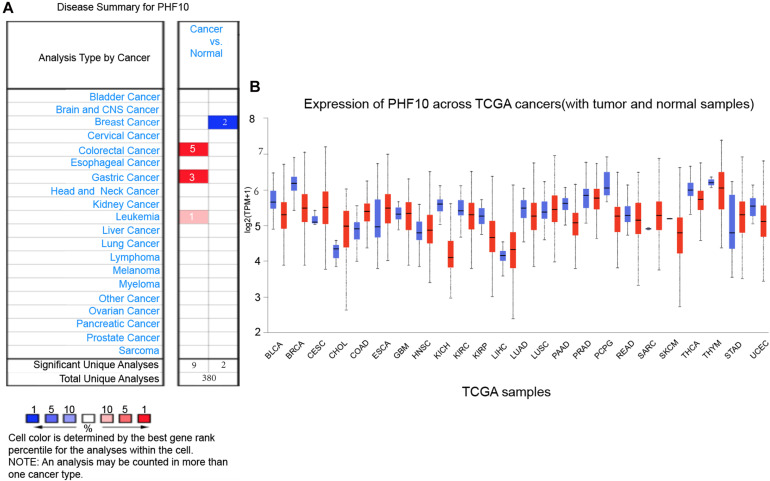
PHF10 mRNA expression in different cancer types, **(A)** mRNA upregulation (Red), and downregulation (Blue) of PHF10 in different cancer types retrieved from the ONCOMINE database. The data were selected based on *P*-value: 1E-4, fold change: 2, and top 10% gene rank. PHF10 is upregulated in Gastric Cancer which is indicated in Red color. **(B)** Expression of PHF10 across various cancer TCGA Cancer data with tumor (red) and normal samples (Blue) depict as boxplots using UALCAN web that include the values between upper and lower quartile inside the box and dashed lines indicate the upper and lower limit of average expression.

To explore the expression pattern of the final PHF10 signature, we went through comparative expression analysis of PHF10 between different cancer and normal tissues using an online web-server, such as ONCOMINE and UALCAN. ONCOMINE compares the difference in expression level between the cancer tissue and normal tissue depending on the numbers of significant unique analyses, which represents the differences in PHF10 mRNA expression. These results were obtained based on parameters as *p*-value < 1.0E-4, gene ranking in the top 10%, and fold-change > 2. In this analysis, we found the upregulation of PHF10 expression in three different cancer types, including colorectal cancer, GC, and leukemia, and downregulation of PHF10 in breast cancer (Figure 5A). We then used the UALCAN database to analyze the expression levels of the PHF10 signature in 24 types of cancers and normal tissues. PHF10 was upregulated and downregulated in 12 different cancers, respectively (Figure 5B).

**FIGURE 6:**
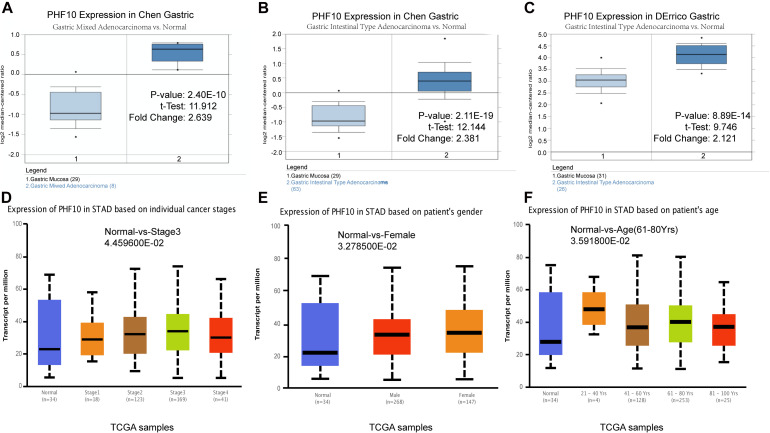
Expression of PHF10 between samples with different subtypes and normal samples from three studies using ONCOMINE database **(A–C)**. Expression of PHF10 in TCGA STAD based on clinicopathological parameters visualized by the UALCAN web server. The analysis was done to predict the expression of STAD and their relative tissue based on, **(D)** stages, **(E)** gender, and **(F)** age.

To understand the PHF10 expression relationship among GC and STAD subtypes, we examined each of the subtype datasets using the ONCOMINE and UALCAN databases. In a detail, we analyzed PHF10 expression in GC as compared to tissue for gastric mixed adenocarcinoma and gastric intestinal type adenocarcinoma. We found a significant upregulation of PHF10 in each GC compared to different control conditions (Figures 6A–C). Furthermore, we examined the expression levels of the PHF10 gene on the basis of various clinicopathological characteristics of GC patients, including Normal (*n* = 34) and different stages: stage 1 (*n* = 34), stage 2 (*n* = 123), stage 3 (*n* = 169), and stage 4 (*n* = 41), gender: Males (*n* = 268) and Females (*n* = 147), age: 21–40 years (*n* = 4), 41- 60 years (*n* = 128), 61–80 years (*n* = 253) and 81–100 years (*n* = 25). For individual cancer stages, we observed the highest expression level of PHF10 in patients with stage 3 (Figure 6D). Additionally, female patients (Figure 6E) and 61–80-year-old age group (Figure 6F) patients were found to express a higher level of PHF10.

## Discussion

This study constructed a GC-specific global network to identify and analyze prognostic signatures using mRNA and ncRNA expression datasets from the TCGA database. During the integrated analysis, we performed three levels of random walk algorithm, which respectively regarded prognostic mRNAs, miRNAs, and lncRNAs as the seed nodes. The final IRW score was calculated for each candidate’s prognostic signature. We also explored the performance of combination signatures, which ranked in the top 100 according to IRW score. Finally, we focused on three key prognostic signatures (miR-22, PHF10, and LINC00592), and explored the expression pattern of PHF10 in GC formation and clinical characterizations. To sum up, we comprehensively analyzed the degree of mRNA effect, lncRNA, and miRNA based on the global network to identify the prognostic signatures of GC.

A number of GC prognostic signatures have been identified to date, based on high-throughput expression data at the single gene-level, miRNA-level, or lncRNA-level. For example, a 7-gene signature associated with the G2/M checkpoint was identified as a prognostic biomarker of GC patients (Lv et al., 2020). Bao et al. (2019) established a 3-gene signature of diffuse type GC for explaining the molecular mechanism of poor prognosis. In another study, a 5-gene signature was established that can be an independent prognostic factor in GC patients (Song et al., 2019). A stromal-immune score-based gene signature was also well-established as a tool for stratification of GC prognosis (Wang H. et al., 2019). Besides gene-based signatures, a number of ncRNA signatures have also been identified. A 6-lncRNA signature was identified by microarray re-annotation of the dataset containing 370 GC samples with clinical information. It was reported that four lncRNAs contributed to GC development, and one lncRNA might be associated with the prognosis of GC (Ma et al., 2020). Another study indicated that lncRNA MNX1-AS1 was a prognostic indicator for GC patients (Ma et al., 2019).

Compared with a single level, the optimization analysis of multiple levels based on a global network displayed more advantages. For GC research, some ceRNA networks were constructed for exploring tumor biological mechanisms. For example, Arun et al. (2018) profiled 19 cancer-associated lncRNAs in thirty gastric adenocarcinomas using qRT-PCR, and further identified risk genes or miRNAs which were correlated with these lncRNAs. Finally, they constructed an integrated lncRNA-miRNA-mRNA interaction network to understand the complex gene regulatory mechanism (Arun et al., 2018). In another study, Zhang et al. adopted the WGCNA method to identify GC related networks. A ceRNA network that included 86 dysregulated relationships was also constructed for simple display (Zheng et al., 2020). In many other tumor types, a similar integrated analysis was also performed. For example, Wang Y. et al., 2019 constructed a ceRNA network to reveal the core ceRNAs in endometrial cancer. Fan et al. (2018) constructed a breast cancer ceRNA network and developed a risk score model based on lncRNAs using multivariate cox regression. A similar framework was also performed on colorectal cancer, and LASSO analysis was utilized to calculate the coefficients for the risk model (Xiong et al., 2017). Compared with these studies mentioned above, our study performed three-level topology analysis based on global ceRNA network respectively using miRNA-, lncRNA- and gene-level seed nodes, which displayed more advantages than simple ceRNA analysis.

To test the biological functions of prognostic seed nodes, we performed functional enrichment analysis based on protective and risk genes (see Figure 2B). Many of the GO terms identified were associated with the formation or progression of GC. Studies have shown that the PI3k/Akt/mTOR pathway is activated in 30%60% of GC. Numerous clinical trials have been conducted by using single or dual Akt/mTOR inhibitors for targeted therapy (Matsuoka and Yashiro, 2014; Tapia et al., 2014). The previous study demonstrated that the ERK/MAPK pathways were involved in a variety of human tumor types, including GC (Guo et al., 2020). In GC patients, high levels of TGF-β1 in serum and cancer tissues were associated with poor prognosis (Fu et al., 2009). Chen et al. (2019) found that gene silencing inhibited the migration and invasion of GC cells by regulating the activation of the TGF-β signaling pathway. At the miRNA level, most prognostic miRNAs were also involved in the GC biological mechanism. A previous study showed that members of the miRNA-29 family can inhibit cell proliferation, and invasion of GC cells by silencing different key targets (Kwon et al., 2019). The dysregulation of miR-125a and miR-125b can affect prognosis and further regulated gastric cell proliferation and apoptosis (Yang et al., 2013, 2019). Deng et al. revealed that miRNA-34a served as a cancer suppressor gene (Zhang et al., 2009). Diffuse manifestations of low miR-200b were related to EMT in patients with GC. All these studies confirm the close association between random walk seed nodes and GC biological events.

After multiple-level random walk analyses, we further calculated the IRW score for each candidate signature and the signature with a higher score was regarded as GC prognostic signature. MiR-22 was ranked top one in all candidates, and the regulatory roles of miR-22-3p were confirmed by the proliferation and apoptosis of GC cells (Li et al., 2020). MiR-125a (ranked second) restrained cell migration and invasion by targeting STAT3 in GC Cells (Yang et al., 2019). DOS revealed that the downregulation mechanism of miR-125a-5p expression in gastric diseases is a potential marker for the early diagnosis of GC (Dos Santos et al., 2020). In addition, miR-126 (ranked third) has been identified as an indicator of poor prognosis and recurrence in GC, especially in patients with histologically negative lymph nodes (Feng et al., 2018). *In vitro*, miR-29a (ranked fourth) could inhibit the growth and invasion of GC cells (Chen et al., 2014). The SNP of miR-184 (ranked fifth) binding-site in TNFAIP2 was related to risk of GC (Xu et al., 2013).

**FIGURE 7:**
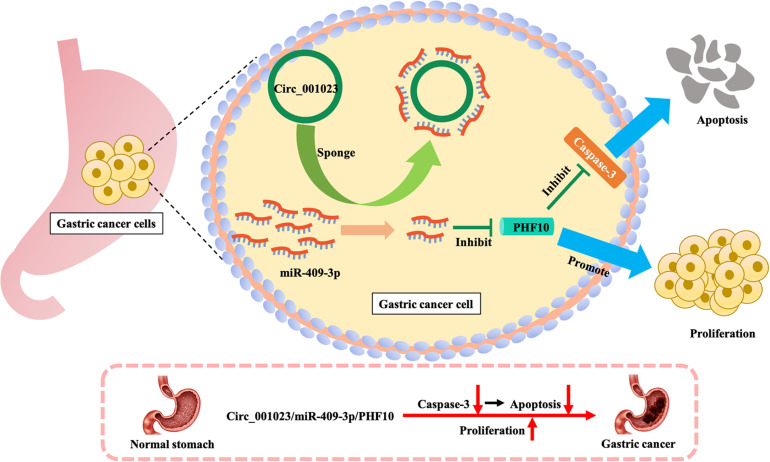
Mechanism of promoting proliferation and inhibiting apoptosis of gastric cancer cells by PHF10.

Based on the IRW score and robustness analysis, we focused on a novel gene signature, PHF10 (plant homeodomain finger protein 10), which belongs to the zinc finger protein family. A previous study (Wei et al., 2010) validated the protein expression levels of PHF10 in GC cell lines, which were significantly higher than those in a normal gastric epithelial cell line. The apoptosis of GC cell line was significantly increased after PHF10 was knocked down, which was consistent with the roles of PHF10 in GC tissues detected in the Oncomine database (see Figures 5, 6A–C). In another study (Li et al., 2012), the regulatory relationship between miR-409-3p and PHF10 was confirmed. This study performed a series of experiments to show that miR-409-3p was involved in the gastric cell growth, apoptosis, and tumorigenesis processes. According to our findings, miR-409 was the only miRNA targeting PHF10 and the second robust signature in combination analysis (see Figure 3C). Furthermore, another regulatory circRNA on the miR-409/PHF10 axis was identified and the complex correlated relationship between them was explored, further showing the biological reliability of the PHF10 signature in GC (Wang et al., 2020; see Figure 7).

We performed systematically integrated analyses using high-throughput mRNA, lncRNA, and miRNA expression profilings from TCGA, and further identified several candidate prognostic signatures based on a multiple-level global network optimization analysis. Future studies will explain the intrinsic biological significance of these signatures further. The findings of the present study are potentially useful for understanding GC prognostic events and identifying robust risk signatures for guiding clinical treatment.

## Data Availability Statement

The TCGA-STAD datasets including the expression datasets and the patients’ clinical information was downloaded from the TCGA database (URL) at https://portal.gdc.cancer.gov/. The validation gastric cancer mRNA expression matrix with available patient survival information was downloaded from GEO database under the accession number GSE38749 in https://www.ncbi.nlm.nih.gov/geo/.

## Author Contributions

SJ conceived and designed the experiments. LC, PW, DN, JS, GT, DL, XZ, WM, and CZ analyzed the data. LC and PW wrote the manuscript. All authors read and approved the final manuscript.

## Conflict of Interest

The authors declare that the research was conducted in the absence of any commercial or financial relationships that could be construed as a potential conflict of interest.

## Footnotes

1 https://cancergenome.nih.gov/

2 https://www.ncbi.nlm.nih.gov/geo/

## Supplementary Material

Supplementary Figure 1 The Gene Ontology (GO) enrichment analysis results for risk genes **(A)** and protective genes **(B)**.
