# Supplementary figures and images for: Identification of a Novel Prognostic Signature for Gastric Cancer Based on Multiple Level Integration and Global Network Optimization
Source: Front Cell Dev Biol. 2021 Apr 12;9:631534. doi: 10.3389/fcell.2021.631534 (PMC8072341; doi:10.3389/fcell.2021.631534)

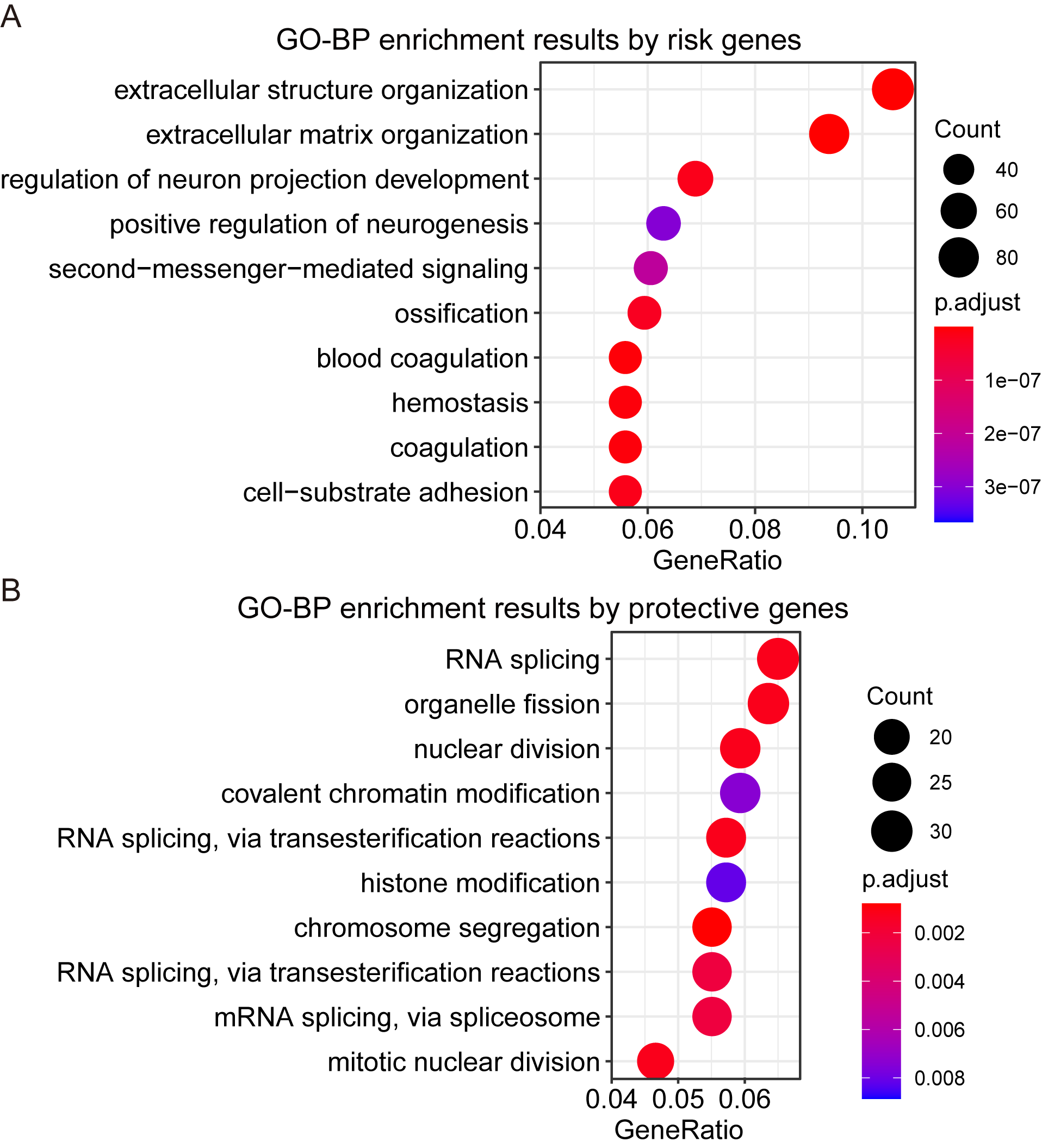

Supplement: Supplementary Figure 1 — The Gene Ontology (GO) enrichment analysis results for risk genes (A) and protective genes (B). [file Image_1.TIF]
